# Inorganic Janus particles for biomedical applications

**DOI:** 10.3762/bjnano.5.244

**Published:** 2014-12-05

**Authors:** Isabel Schick, Steffen Lorenz, Dominik Gehrig, Stefan Tenzer, Wiebke Storck, Karl Fischer, Dennis Strand, Frédéric Laquai, Wolfgang Tremel

**Affiliations:** 1 Institut für Anorganische Chemie und Analytische Chemie, Johannes Gutenberg-Universität, Duesbergweg 10–14, 55128 Mainz, Germany; 2 Medizinische Klinik und Polyklinik, Universitätsmedizin der Johannes Gutenberg-Universität, Langenbeckstrasse 1, 55131 Mainz, Germany; 3 Max-Planck-Institut für Polymerforschung, Max-Planck-Forschungsgruppe für Organische Optoelektronik, Ackermannweg 10, 55128 Mainz, Germany; 4 Institut für Physikalische Chemie, Johannes Gutenberg-Universität, Jakob-Welder-Weg 11, 55128 Mainz, Germany

**Keywords:** bioimaging (CT, MRI, Multi-photon), hetero-nanoparticles, Janus particles, protein corona, synthesis

## Abstract

Based on recent developments regarding the synthesis and design of Janus nanoparticles, they have attracted increased scientific interest due to their outstanding properties. There are several combinations of multicomponent hetero-nanostructures including either purely organic or inorganic, as well as composite organic–inorganic compounds. Janus particles are interconnected by solid state interfaces and, therefore, are distinguished by two physically or chemically distinct surfaces. They may be, for instance, hydrophilic on one side and hydrophobic on the other, thus, creating giant amphiphiles revealing the endeavor of self-assembly. Novel optical, electronic, magnetic, and superficial properties emerge in inorganic Janus particles from their dimensions and unique morphology at the nanoscale. As a result, inorganic Janus nanoparticles are highly versatile nanomaterials with great potential in different scientific and technological fields. In this paper, we highlight some advances in the synthesis of inorganic Janus nanoparticles, focusing on the heterogeneous nucleation technique and characteristics of the resulting high quality nanoparticles. The properties emphasized in this review range from the monodispersity and size-tunability and, therefore, precise control over size-dependent features, to the biomedical application as theranostic agents. Hence, we show their optical properties based on plasmonic resonance, the two-photon activity, the magnetic properties, as well as their biocompatibility and interaction with human blood serum.

## Introduction

**Figure 1:**
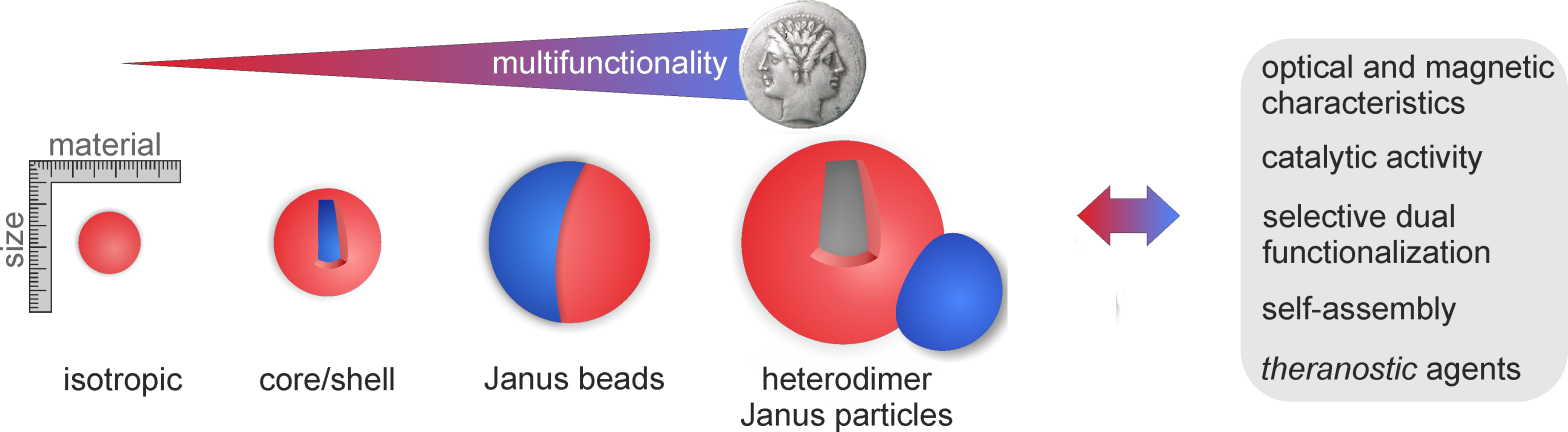
Illustrations of the transition from isotropic to anisotropic particles.

In the recent years, there has been an increasing interest in design, synthesis, and properties of multifunctional nanoparticles owing to their special structure–property relationship [1]. Due to their distinct surface properties, these nanomaterials can be modified by various ligands to introduce the desired surface characteristics ranging from solubility in selected solvents [2], specificity towards small molecules or larger biomolecules [3], suppression of nonspecific adsorption [4], adjustment of net electric charge [5], to electrochemical activity [6]. Although it has been shown that synthetic and natural systems share a number of similarities, the degree of asymmetry in building block structures and also in their interactions is quite different. While the huge majority of synthetic building blocks has (centro)symmetric morphology and interaction potentials (e.g., Coulomb interaction), this is rarely found in natural systems. Therefore, the introduction of complexity through asymmetry is a major challenge in synthetic nanochemistry (Figure 1). Hetero-nanoparticles facilitate an even more advanced approach toward the design of today’s highly desired multifunctional nanoparticles used in research at the interface between materials science, biotechnology, and medicine [7–8]. Therefore, the enhancement of nanoscaled properties is obtained through synergistic interactions between the single components as well as the designed evolution of additional properties, which are not realizable in single component nanoparticles [9].

Hetero-nanoparticles enable access to widespread technological scenarios. These hybrid materials represent artificial platforms generating synergistically enhanced, tunable chemical and physical characteristics, or even cause the emergence of phenomena, which would not be accessible using homogeneous nanomaterials.

**Figure 2:**
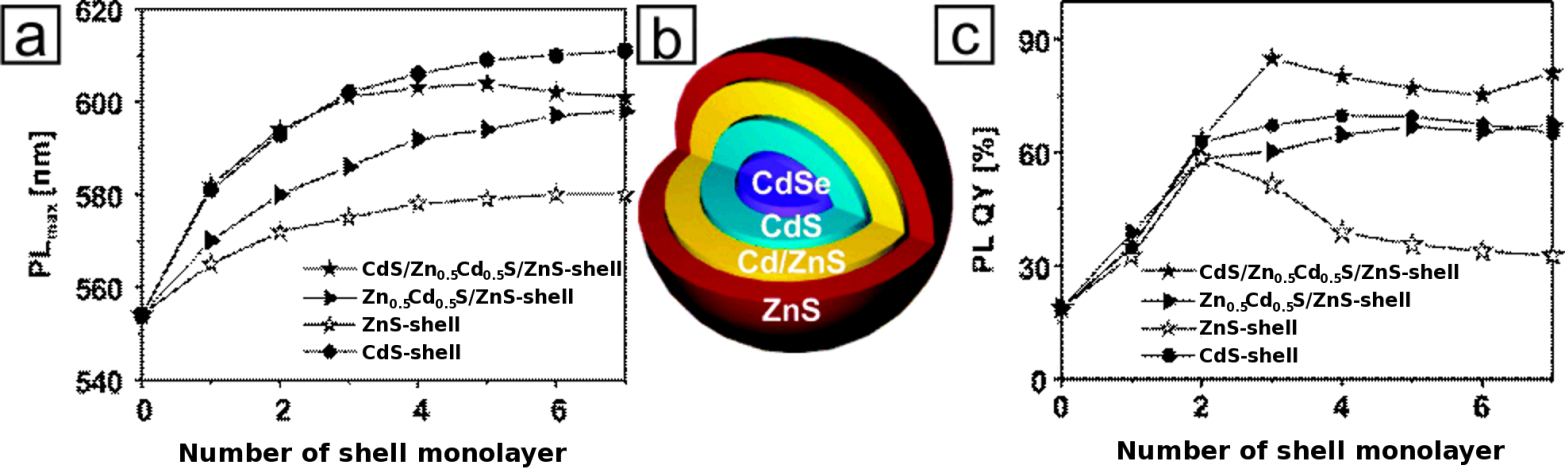
a) Evolution of the PL-peak position, b) schematic representation, and c) evolution of the PL-quantum yield for several core-shell quantum dots starting with a CdSe core. Adapted with permission from [13]. Copyright 2005 American Chemical Society.

When the inorganic core contains two or more chemical species with a combination of metals, metal oxides, or chalcogenides, the composition and distribution of each of those phases are crucial parameters in addition to crystal structure, size, and shape. The most commonly studied morphology of inorganic hetero-structured nanoparticles are core/shell structures, in particular core/shell structures of fluorescent II–IV and III–V semiconductors, typically transition metal-chalcogenides, -phosphides, and -arsenides [10]. The epitaxial combination of a 0D spherical quantum dot with a 1D rod-like shell of a semiconductor leads to enhanced optical characteristics such as high luminescence quantum yields [11–12], decreased fluorescence, as well as tunable emission color as a function of particle size and shell thickness. This optimization is governed by the depression of structural imperfections at the surface and the enhanced chemical robustness as compared to single-component analogues [13–15]. Thus, by an appropriate choice of structure and composition, the epitaxial combination can be applied to enable band engineering resulting in hetero-nanostructures with unconventional behavior regarding their optoelectronic properties [16–17]. Since the 1980s [16,18–19], there has been a huge research effort in optimizing the characteristics of hetero-structured semiconductors with extraordinary optical properties. Xie et al. [13] demonstrated the design of CdSe quantum dots covered by a multishell structure from CdS and ZnS by using the successive ion layer adhesion and reaction (SILAR) technique (Figure 2b). Upon the gradual change of the composition in radial direction, a bathochromic shift of the photoluminescence-band was observed due to the reduced confinement (Figure 2a). This goes along with the enhanced quantum yield governed by the electronic passivation of the surface, whereas the quantum yield was decreased for a shell thickness of more than two multilayers, which was attributed to lattice imperfections within the shell (Figure 2c) [13].

Recent developments in the fields of hetero-structured quantum dots are the formation of anisotropic, namely elongated structures, whereby the variation of the aspect ratio allows control of the electron–hole overlap. This leads to an exceptional size-dependent quantum Stark effect due to the increased spatial volume enabling effective charge carrier separation [20]. The design of anisotropically shaped semiconductors is limited since the band-edge luminescence is further reduced due to a higher surface-to-volume ratio and the increased carrier delocalization lowering the probability of a radiative carrier recombination [10], but their major drawback, the uniformity of the surface, remains.

## Review

### Janus particles as next generation nanoparticles

**Figure 3:**
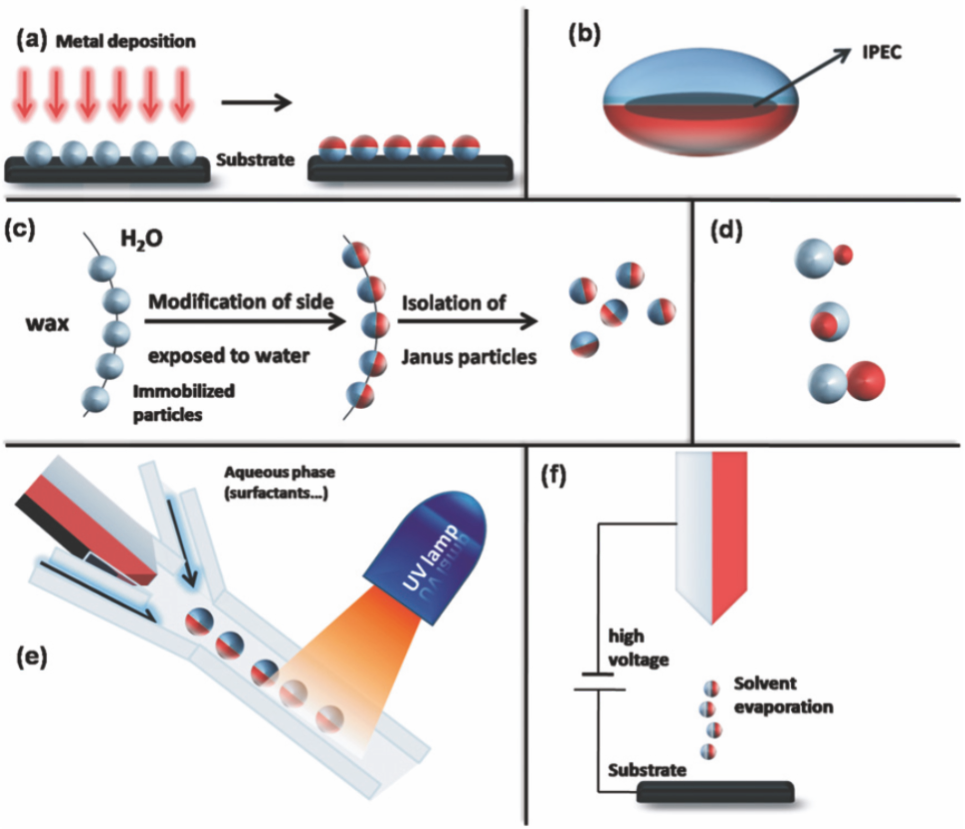
Summary of synthetic routes towards organic Janus particles. (a) Directed functionalization after immobilization, (b) ellipsoidal complex core coacervate micelle with an inter-polyelectrolyte complex core (IPEC), (c) classical Pickering emulsion technique, (d) different topologies of Janus particles: snowman-, acorn-, and dumbbell-like nanoparticles (top to bottom), (e) microfluidic photo-polymerization system, and (f) electrospinning technique with a bi-phasic nozzle. Reproduced with permission from [29]. Copyright 2008 The Royal Society of Chemistry.

Janus particles are outstanding among the hetero-nanoparticles owing to their asymmetry as an additional design module. Therefore, they are able to combine even very different chemical and physical properties within a single particle. De Gennes was one of the first to point out the significance and potential of Janus particles in his Nobel lecture entitled “Soft Matter” in 1991 [21]. Eponym is the two-faced Roman god Janus, the god of beginning and ending, doors and gates [22]. Initiated by the lecture of de Gennes, a huge research effort was put onto the design of Janus structures starting with the first Janus particles in the early 1980s by Veyssié and co-workers [23]. Their approach to “Janus pearls” was the deposition and immobilization of particles to a surface and subsequent functionalization of the exposed surface by a metal (Figure 3a). A major drawback is that the method is restricted to small amounts of nanoparticles and cannot easily be up-scaled. Nevertheless, the method is still used because of its simplicity and extended to liquid–liquid interfaces of Pickering emulsions to obtain spatial control over functionalization [24]. A classic example for the directed functionalization of isotropic silica particles is the use of nanoparticles adsorbed to a solidified wax–water interface, where they are selectively exposed to silane vapor. This principle can be applied to create amphiphilic or dipolar Janus particles [25]. The effectiveness of this technique of selective functionalization was shown by Perro and co-workers applying a Pickering emulsion of wax-in-water to obtain a large amount of Janus silica particles with a diameter below 100 nm (Figure 3c) [26].

Nevertheless, dumbbell-shaped nanoparticles provide enormous advantages over spherical, desymmetrized nanoparticles because of the combination of intrinsically different surfaces with distinct reactivity. The design of heterostructured Janus particles began with organic Janus particles composed out of two different polymers forming an amphiphilic particle. Kumacheva et al. reported a microfluidic method for fast continuous synthesis of Janus particles as well as three-phase particles with narrow size distribution by emulsification of monomer liquids and in situ photoinitiated polymerization of multiphase droplets (Figure 3e) [27]. Another approach to polymer-based particles with two distinct phases is the simultaneous electrohydrodynamic jetting of polymer solutions under the influence of an electrical field (Figure 3f) [28].

**Figure 4:**
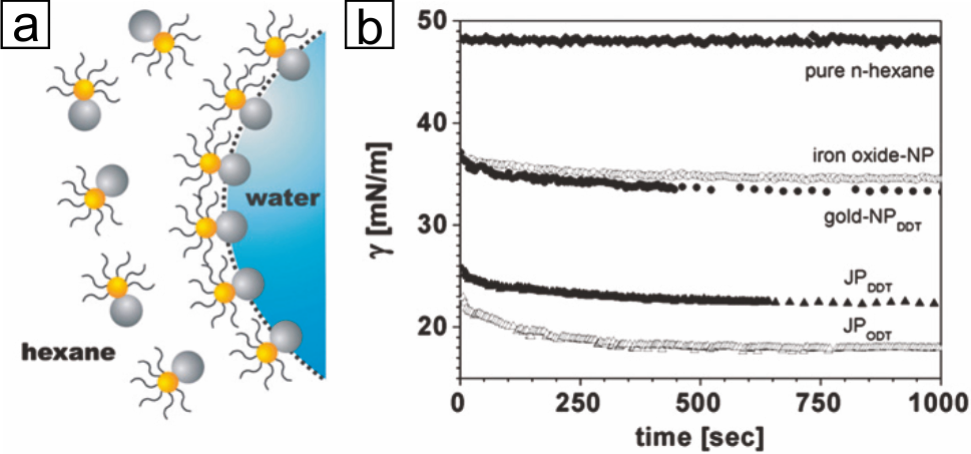
(a) Schematic representation of bimetallic Janus particles at the hexane–water interface (gold: gold part with surfactant; gray: iron oxide part). (b) Interfacial tension vs time as measured by pendant drop tensiometry (NP: homogeneous nanoparticles; JP: Janus particles). The gold domains were functionalized using 1-dodecanethiol (DDT) or 1-octadecanethiol (ODT). Adapted with permission from [32]. Copyright 2006 American Chemical Society.

Further, Janus particles are in focus of current research due to their self-organization into complex and well-defined assemblies, which was found to obey to the same rules as for molecular assembly [30]. The tunability of the surface-active properties of particles with a segregated corona over particles with a uniform wettability enables access to an even greater extent of asymmetry, as known from natural building blocks, and makes these particles of particular importance. Binks and co-workers [31] predicted a strengthened adsorption at an oil–water interface due to the increase in surface activity by a factor of three of a Janus. Glaser et al. [32] demonstrated that Au@Fe_3_O_4_ Janus particles reduce the interfacial tension of the oil/water interfacial significantly compared to similar uniform particles, thus confirming previous theoretical predictions (Figure 4). Furthermore, the interfacial activity is enhanced by increasing the amphiphilic character using long alkyl chain thiols.

**Figure 5:**
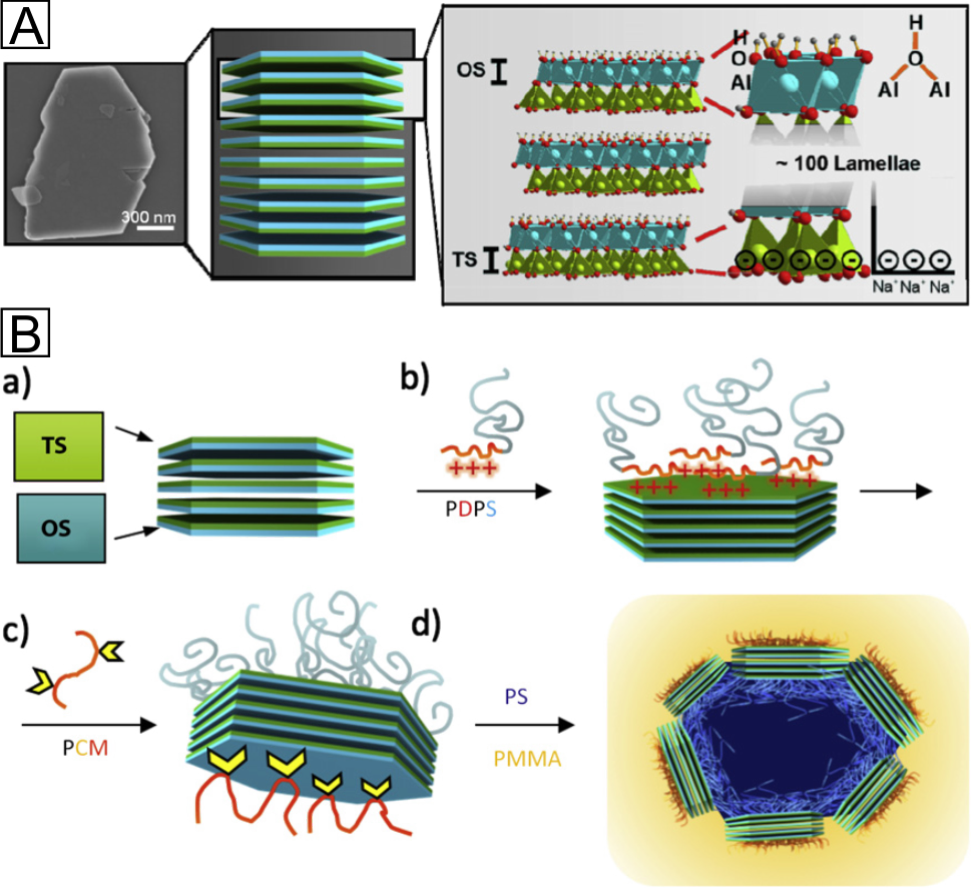
(A) SEM top view image of a typical kaolinite platelet (left), schematic picture of kaolinite platelets (centre), and crystal structure of three kaolinite lamellae with the specific chemical functions at the basal TS and OS (right). (B) Schematic picture of a) pristine kaolinite, b) modified with PDPS on the tetrahedral surface (TS), c) further modified with PCM on the opposite octahedral surface (OS), and d) embedding of the final hybrid particle at the interface in a PS-PMMA blend. Reprinted with permission from [34]. Copyright 2013 Elsevier.

The theme of (self-assembled) Janus building blocks is inspired by nature where it is most common present in form of organic materials such as lipids or proteins, amongst others a class of fungi proteins called hydrophobins. Moreover, Breu and co-workers recently demonstrated the native Janus character of the natural mineral kaolinite [Al_2_Si_2_O_5_(OH)_4_], which is a dioctahedral layered silicate found in form of anisometric platelets with large aspects ratios (Figure 5). Due to the hydrophilic nature of both surfaces, the Janus character remains hidden until the octahedral and tetrahedral surfaces are selectively modified by cation exchange and covalent grafting of catechol ligands. Up to now, large technical applications of Janus particles are restricted by the lack of accessibility. Therefore, polymer-modified kaolinite provides the enormous advantage as an abundant, ubiquitous, and inexpensive mineral, which can be used as superior Pickering emulsifier [33–34].

### Inorganic Janus particles

Based upon the combination of optical, magnetic, and catalytic properties within one nanoparticle, inorganic hybrid materials have attracted increasing attention owing to their easily tunable properties by variation of materials, domain sizes, and morphology. Aside from the properties of the single components added one by one to form the properties of the heterostructure, several new properties emerge from the morphology and surface chemistry of the heterodimers [35]: (i) multifunctionality based on the different surface chemistry of the chosen components enabling separate and selective surface functionalization [36–39] in addition to the intrinsic multifunctionality due to the combination of the two inorganic components (e.g., combination of optical and magnetic properties), (ii) directed self-assembly, achieved by orthogonal functionalization of the surfaces [30,40–41]. In addition to the extrinsic characteristics arising from exploiting the anisotropy, efficient charge separation [42], magnetic interaction [43], or spin-polarization transfer [44] at the interface of the hetero-nanoparticle can be realized by heterodimer nanoparticles. The epitaxial interaction of two components was shown to change or enhance the characteristics, or even create new properties compared to single component nanoparticles. Regarding catalytic activity, the metal oxide domain acts as charge reservoir, while the metal domain acts as active component toward metal-organic reactions. For instance, this enhanced catalytic activity in comparison to the single component nanoparticles was demonstrated for Ni@Fe_2_O_3_ [45] or Pt@Fe_3_O_4_ [46]. Furthermore, the magnetic anisotropy and coercivity of Fe_3_O_4_ was significantly increased due to conjugation to Ag nanoparticles when combined to form Ag@Fe_3_O_4_ dumbbell-like hetero-nanoparticles [47].

**Figure 6:**
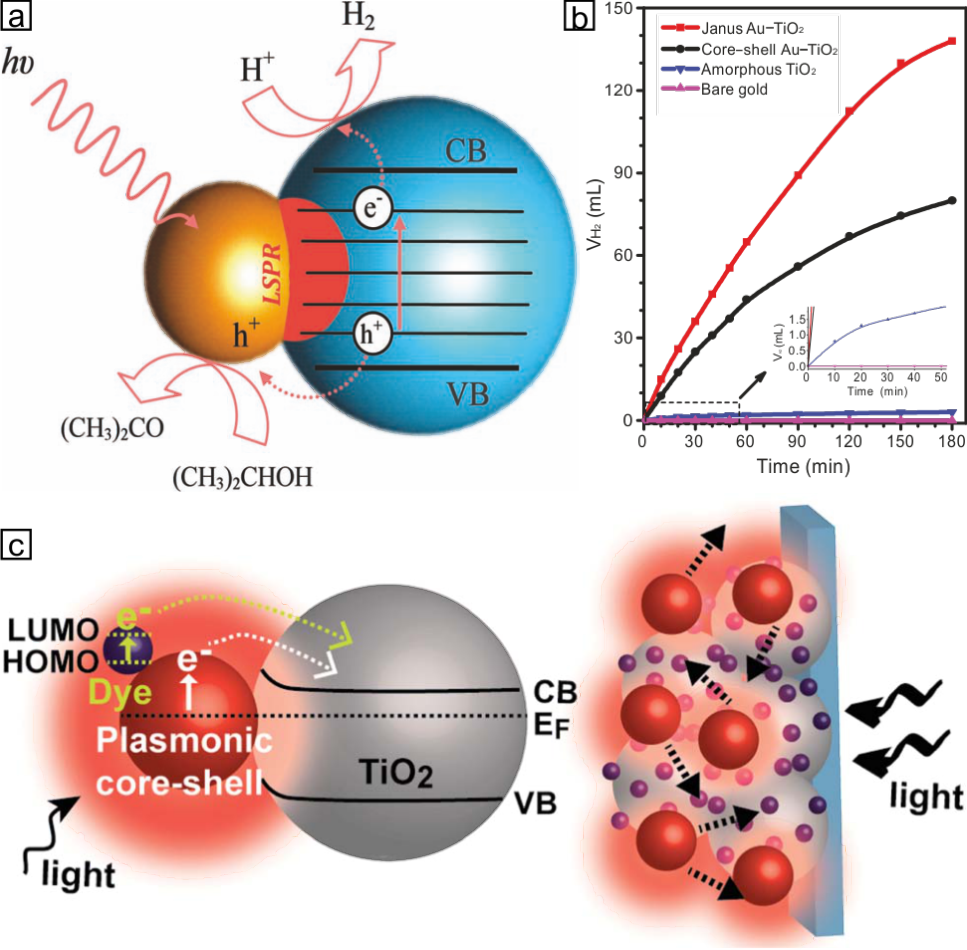
a) Proposed photocatalytic process for efficient hydrogen generation using the Janus Au@TiO_2_ nanostructures, based on excitation of the LSPR under visible-light irradiation [50]. b) Volume of hydrogen generated (*V*_H2_) under visible-light irradiation from a tungsten halogen lamp using Janus and core-shell Au_50nm_@TiO_2_ nanostructures, as well as amorphous TiO_2_ and bare gold particles [50]. c) Schematic illustration of plasmonic dye-sensitized solar cells (DSSCs) with tailor-designed Au–TiO_2_ nanostructures integrated into the photoanode representing the increased photocurrent by LSPR and scattering effects [51]. (a, b) Adapted with permission from [50]. Copyright 2012 WILEY-VCH. (c, d) Reproduced with permission from [51]. Copyright 2014 The Royal Society of Chemistry.

Moreover, plasmonic photocatalysts combine two prominent features: a Schottky junction enhancing charge separation and surface plasmon resonance, which is responsible for strong absorption of visible light und the excitation of charge carriers within the photocatalyst [48]. Upon conjugation of semiconductor nanoparticles, such as TiO_2_ to metal nanoparticles, charge equilibration takes place when the composite material is photoexited (Figure 6c). As a direct consequence, the Fermi level of semiconductor nanoparticles is shifted to more negative potentials, thus, enabling the engineering of the Fermi level of photocatalysts dependent on the size of the conjugated metal domain [49]. Recently, Au@TiO_2_ Janus particles were proven useful for visible-light hydrogen generation due to the strong coupling of plasmons to the optical transitions in amorphous TiO_2_ leading to enhanced optical absorption and, thus, generation of electron–hole pairs for photocatalysis (Figure 6a,b) [50]. Furthermore, plasmonic dye-sensitized solar cells based on Au@TiO_2_ nanostructures show remarkably enhanced power conversion efficiencies. This influence was mainly ascribed to the enhanced dye absorption by the magnified near-field of Au nanoparticles and the plasmon-enhanced photocurrent generation (Figure 6c) [51].

### Synthesis of inorganic Janus particles

The synthesis of hetero-nanoparticles requires an even higher degree of synthetic control as compared to single-component nanoparticles. Although, some major problems have been overcome by intelligent design of the synthetic procedure within the last decade, the synthesis of well-defined Janus particles specially designed for applications is a continuing endeavour. The formation takes place in the “twilight zone” of kinetic and thermodynamic control, whereby a precise balance is needed for the formation of the desired size and shape. The evolution is further complicated by the interplay of atomic diffusion and exchange, facet-specific reactivity, or the influence of interfacial strain [10].

The main synthetic routes for obtaining inorganic dumbbell-like heterostructures include heterogeneous nucleation [52–53], asymmetric modification at interfaces [41], and non-epitaxial deposition on the full surface of the first nanoparticle followed by thermal dewetting of the shell into a single domain [54]. During the last years, non-hydrolytic approaches have been used to overcome the problems appearing in hydrolytic syntheses such as poor crystallinity, polydispersity in size, and little control over the morphology. The studies presented in the literature so far provide evidence of an enormous progress regarding the efficient synthesis of a plethora of pseudobinary metal–metal oxide hetero-nanoparticles [35,52], such as Pt@Fe_3_O_4_ [46], Pd@Fe_3_O_4_ [55], Au@Fe_3_O_4_ [36–38,56], Ag@Fe_3_O_4_ [47], Cu@Fe_3_O_4_ [57], FePt@MnO [43], Au@MnO [39,58], Ni@Fe_2_O_3_ [45], and Co@Fe_2_O_3_ [59]. These inorganic Janus particles consisting of two different core materials are obtained either via a seed-mediated route using performed seeds or as a one-pot synthesis. However, in both cases the heterogeneous nucleation of a second or third component is taking place, limiting these techniques to material combinations where epitaxial growth is possible [7,60].

In order to create hetero-nanoparticles, it is crucial to suppress homogeneous nucleation of the second (or third) component as competitive reaction to heterogeneous nucleation on the preformed or in situ formed seeds. Following classical theory of heterogeneous nucleation, this can be achieved by decreasing the concentration of the precursor below supersaturation, at which the homogeneous nucleation would be favourable [61]. Furthermore, the additional term of Gibbs free energy for the adhesive energy at the interface between the seeds and the overgrown particles has to be negative, as given for epitaxial growth [56,60].

Lattice mismatch is not only responsible for the preference of a hetero-nanostructure, it also controls the product morphology through the extent of lattice mismatch. Nucleation and epitaxial growth is non-restricted for small differences in lattice constants. For large differences, however, the growth is confined to distinct crystal planes by minimizing the lattice mismatch, which in turn leads to the formation of anisotropic structures [54,62]. At the same time, despite lattice match, it is possible to distinguish between phase separated hetero-nanostructures and ternary phases, when a sufficient molecular control during synthesis is possible and the ternary phases exist according to the phase diagrams [45,57,59].

### Synthesis of dumbbell-like nanoparticles

**Figure 7:**
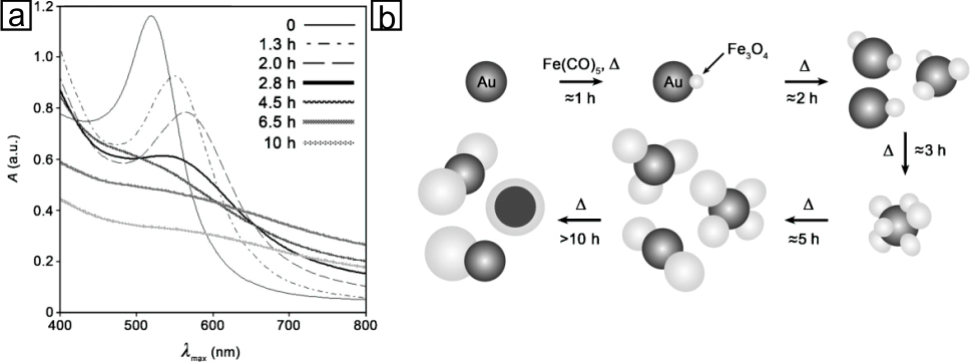
a) UV–vis spectra of Au@Fe_3_O_4_ nanoparticles corresponding to schematic representations in b). The scheme illustrates the shape evolution of the particles during heating (Au: dark gray, Fe_3_O_4_: bright gray). Reproduced with permission from [72]. Copyright 2008 WILEY-VCH.

The most prominent example of inorganic Janus particles used for catalysis [52,63–64], drug delivery [65], bimodal bioimaging [36,66–68], and biomedical applications [69–70] such as cancer treatment [71] are dumbbell-like Au@Fe_3_O_4_ nanoparticles. As no ternary Au-Fe-O phase or a gold oxide is present under the experimental conditions, there is no need to pay attention to achieve phase-separation when synthesizing Au@Fe_3_O_4_ heterodimers, but the morphology control remains a challenge. Wei and co-workers demonstrated the formation of dumbbell to flowerlike Au@Fe_3_O_4_ heterodimer nanoparticles by increasing the ratio of multiply twinned Au seeds to Fe(CO)_5_ with 2 to 6 Fe_3_O_4_ domains [72]. Additionally, the prolongation of reaction time leads to an increasing size of the Fe_3_O_4_ domains (Figure 7). Due to the small lattice mismatch at the interface of 2d_111_-(Au) and d_111_-(Fe_3_O_4_), heterogeneous nucleation is favored and can even be directly monitored by optical spectroscopy [56,73].

**Figure 8:**
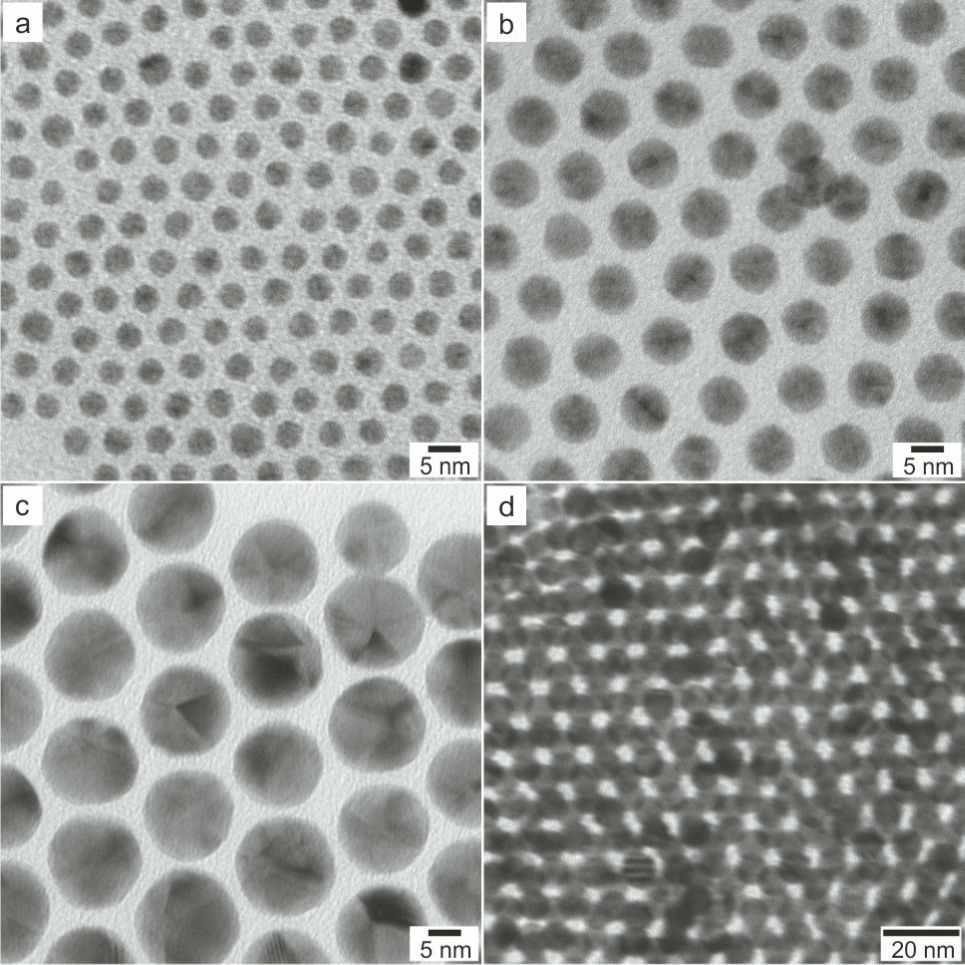
TEM bright field images of Au nanoparticles with different diameters (a) 4 nm, (b) 8 nm, and (c) 15 nm; (d) 2D superlattice of 8 nm Au nanoparticles.

The synthetic route can easily be applied to other material compositions as previously demonstrated by the formation of heterostructures composed out of gold and manganese oxide [39]. Au@MnO heterodimers were prepared using preformed Au nanoparticles as seeds, as the control over the size was much more precise performing a synthesis of hydrophobic gold nanoparticles using *tert*-butylamine borane complex as reducing agent [74]. The size of the oleylamine-capped particles is varied between 2 and 8 nm by changing the reaction temperature or in a subsequent growth reaction to create larger nanoparticles by addition of HAuCl_4_ and oleylamine to preformed gold particles (Figure 8).

**Figure 9:**
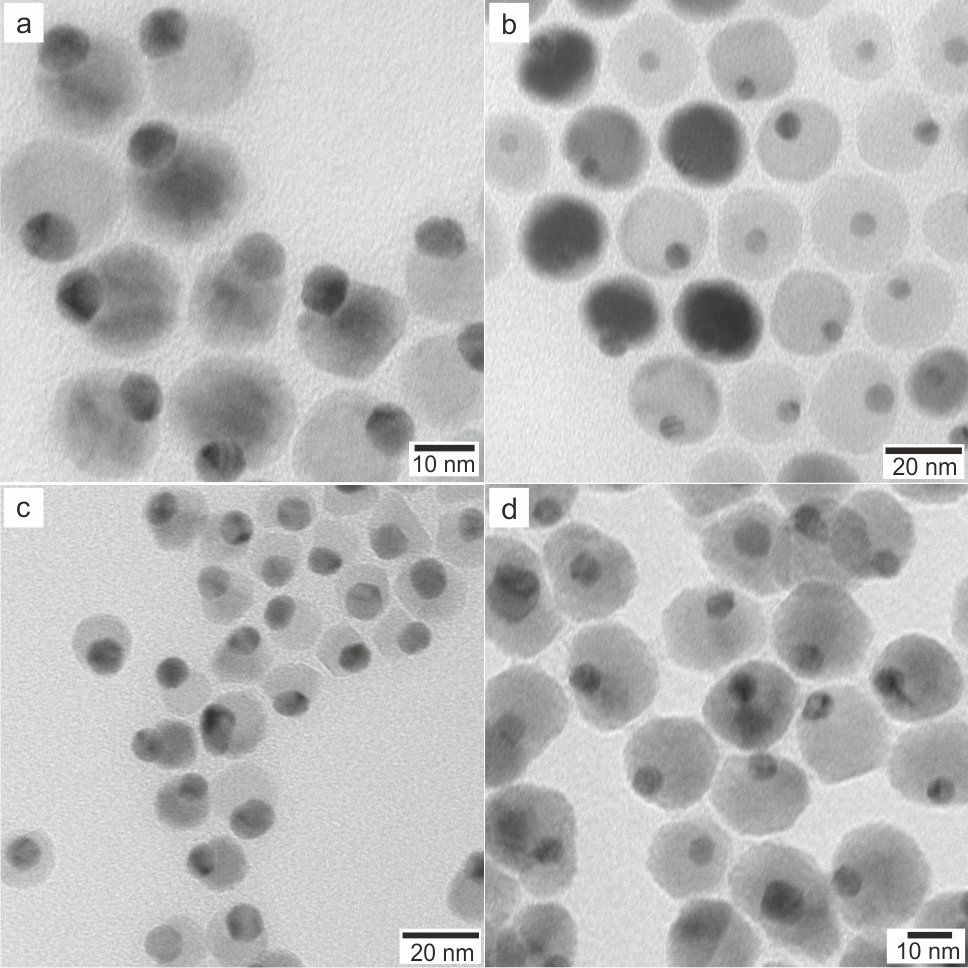
TEM bright field images of Au@MnO and Au@Fe_3_O_4_ heterodimer-nanoparticles: (a) 9@18 nm Au@MnO, (b) 4@22 nm Au@MnO, (c) 9@15 nm Au@Fe_3_O_4_, and (d) 7@20 nm Au@Fe_3_O_4_.

As discussed earlier, the heterogeneous nucleation can be promoted by suppression of homogeneous nucleation, which is concentration dependent and, additionally, can be controlled by the choice of the organometallic precursor. In a subsequent step to the formation of gold particles, the hydrophobic oleate-capped metal oxide domains were heteroepitaxially grown on the preformed seeds by adapting the synthetic parameters for the formation of monodisperse, isotropic metal oxide nanoparticles [75–77]. Inspired by the idea of the formation of a complex composed of the metal ion and the ligand, the strategy of direct employment of a metal-surfactant complex was used. For instance, manganese(II)oleate [77] and iron(III)oleate [64,75,78] can be handled easily and safely due to their low toxicity. Moreover, the higher decomposition temperature of metal oleate complexes compared to metal carbonyls, such as Fe(CO)_5_ [37,52,56], amplifies the control over the morphology and, thus, were used for the formation of Au@MnO and Au@Fe_3_O_4_ heterodimers (Figure 9).

As reported previously [39], the gold nanoparticles were functionalized with 1-octadecanethiol to suppress multiple nucleation of manganese oxide on different crystal facets or surface defects. This surface functionalization was proved not to be necessary for Au@Fe_3_O_4_ heterodimers. The morphology as well as the sizes of the metal oxide domains of the heterodimer nanoparticles were adjusted by a precise control of the precursor ratio, the number of seed particles, and the heating profile.

### Optical characteristics of Au@MO_x_ Janus particles

**Figure 10:**
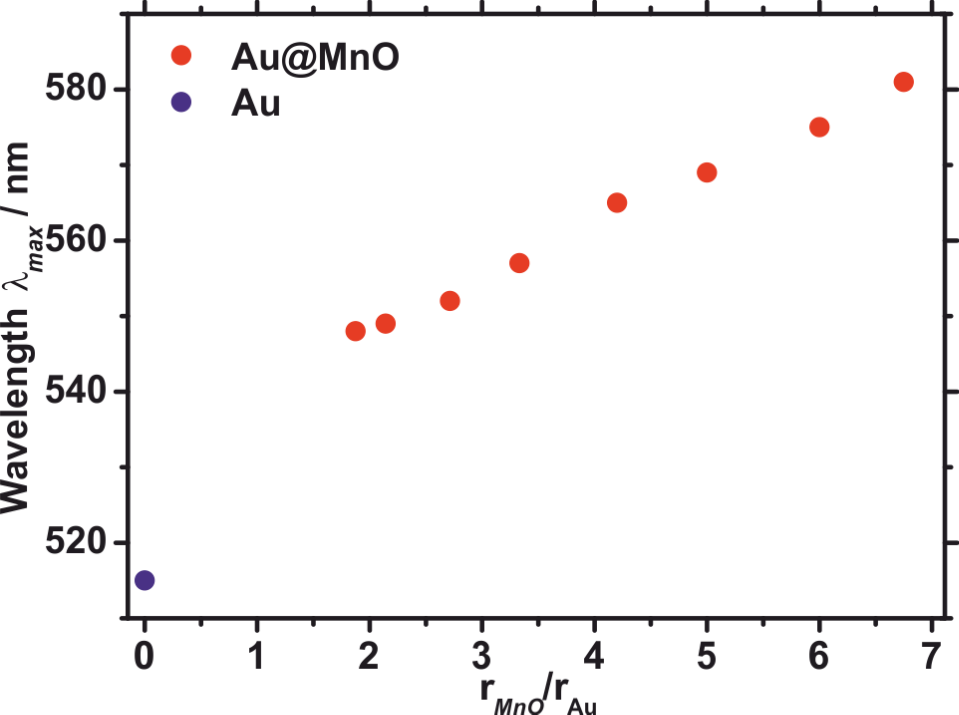
Domain size dependency of absorption maximum of Au@MnO nanoparticles determined by UV–vis spectroscopy in comparison to pristine Au nanoparticles.

Figure 10 shows the dependence of the absorption maximum measured by UV–vis spectroscopy from the domain sizes of Au@MnO heterodimer nanoparticles in comparison to spherical Au nanoparticles. The shift of the absorption maximum amounts to 30 to 60 nm depending on the ratio of the domain sizes of Au and MnO. Mie’s theory describes the direct dependence of the surface plasmon resonance from the local dielectric function surrounding the Au nanoparticles; it is decreased significantly by the conjugation of an electron deficient material such as MnO or Fe_3_O_4_. A different approach utilizes an energy transfer between the gold domain and the metal oxide to explain the bathochromic shift [79–80].

**Figure 11:**
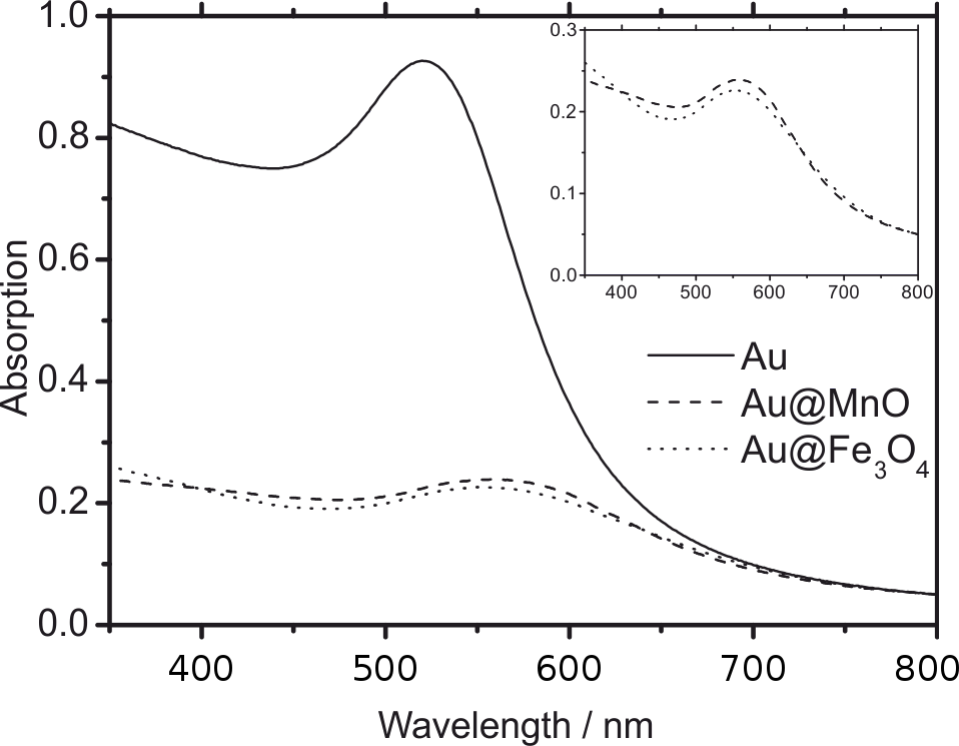
UV–vis spectra of Au (solid), Au@MnO (dashed), and Au@Fe_3_O_4_ (dotted) nanoparticles normalized to the absorption at 800 nm.

On the contrary, Mie’s theory fails to describe the decreased intensity and the broadening of the SPR upon formation of heterodimer-nanoparticles, which is displayed by the comparison of the absorption spectra of pristine Au nanoparticles and Au@MnO and Au@Fe_3_O_4_ heterodimers (Figure 11). Yu et al. attributed this broadening and damping to the tunnelling of conduction band electrons of the Au nanoparticles into the projected density of states of the Fe_3_O_4_ domains, the so-called “interface decay channel” [56]. As a metal oxide starts to nucleate heterogeneously on the gold nanoparticles, the induced charge arising from the polarized plane at the interface is compensated for by the free electrons of the Au nanoparticles. Thus, the remaining gold facets become electron deficient and unsuitable for multiple nucleation. Nevertheless, multiple nucleation can be triggered by adapting the reaction parameters, such as switching to more polar solvents to compensate the electron deficiency for Au@Fe_3_O_4_ flowerlike particles [81], or for Au@MnO flowerlike particles in situ formation of gold particles without surface protection caused by thiol addition [58].

### Phase-separated vs ternary-phase nanoparticles

**Figure 12:**
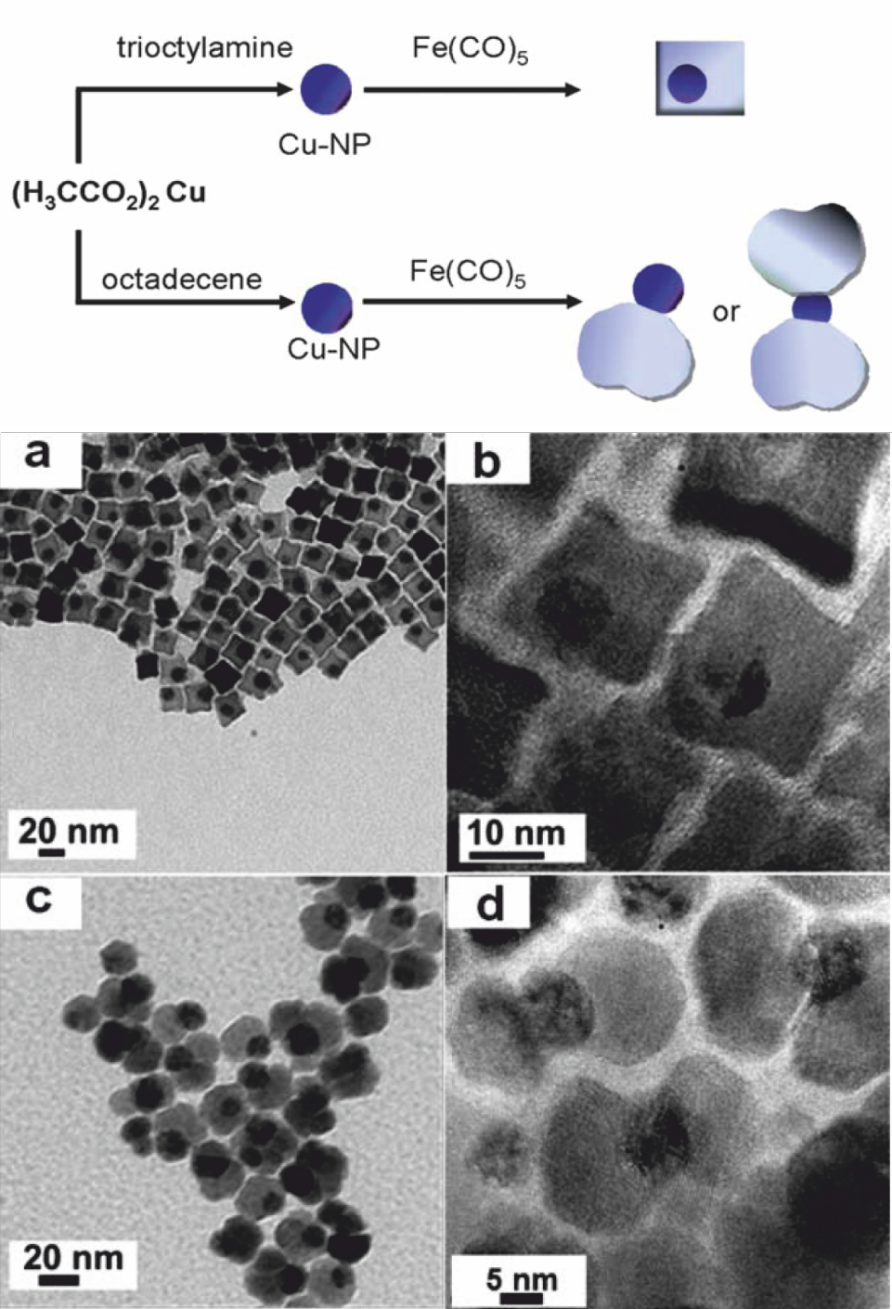
Schematic representation of the formation of Cu@Fe_3_O_4_ heterodimers with different morphologies based on the use of solvents of various polarity (top) and corresponding (HR-)TEM images of (a, b) cube-shape, (c, d) cloverleaf-shape Cu@Fe_3_O_4_ heterodimerparticles (bottom). Reproduced with permission from [57]. Copyright 2011 The Royal Society of Chemistry.

The wet chemical approach for the synthesis of hetero-nanoparticles enables the control of the kinetics of the phase formation and the particle growth via the bottom-up approach. Therefore, solid state diffusion barriers become negligible for phase formation and the sequence of phases is controlled by the decomposition temperatures of the precursors, the relative activation energies for nucleation, as well as the interfacial energies [57,82]. So, even if ternary or at least binary phases are stable under the given experimental conditions, it is possible to synthesize phase-separated hetero-nanoparticles. This was demonstrated for the first time by the formation of Cu@Fe_3_O_4_ heterodimers [57], which was an unexpected result due to the high stability of binary copper oxides, e.g., CuO, Cu_2_O, as well as the ternary phases CuFe_2_O_4_, copper substituted Fe_3_O_4_, or CuFeO_2_. In comparison to solid state chemistry, the crucial step of the nanoparticle synthesis is the formation of a metal seed by decomposition of an organometallic precursor prior to the nucleation of the metal oxide. Simultaneous nucleation of both components would have interdiffusion as a limiting factor. Depending on the solvents used for the formation of the heterodimers, cube shape or cloverleaf shape particles were obtained (Figure 12); the iron oxide phase was always Fe_3_O_4_, independent of the domain morphology.

**Figure 13:**
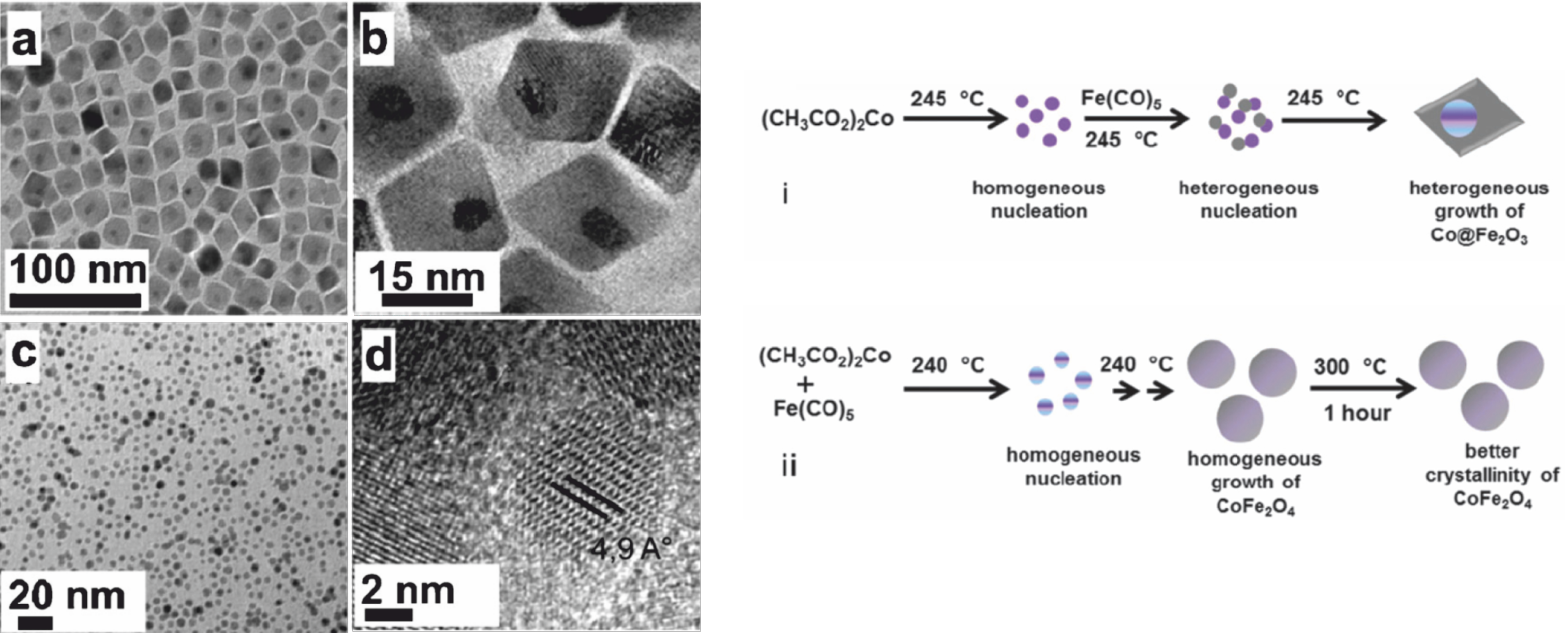
Synthetic protocol of the synthesis of Co@Fe_2_O_3_ heterodimer and phase pure CoFe_2_O_4_ nanoparticles (top) and corresponding (HR-)TEM images of (a,b) heterodimer particles and (c,d) isotropic CoFe_2_O_4_ nanoparticles. Reproduced with permission from [59]. Copyright 2011 The Royal Society of Chemistry.

The wet chemical approach was utilized as well to control the formation of either Co@Fe_2_O_3_ or CoFe_2_O_4_ [59]. As displayed in Figure 13, homogeneous nucleation, facilitated by the subsequent addition of the precursor to the reaction mixture, is absolutely crucial for obtaining phase-separated heterodimers. The ternary metal oxide nanoparticles are obtained under identical reaction conditions, but here all organometallic precursors were mixed simultaneously.

### Janus particles as multimodal contrast agents

Recent developments in the field of nanoparticles for biomedical applications have increased the interest in multifunctional nanoparticles for theranostics, a combination of therapy and diagnostics, which was realized with magnetic nanoparticles in the late 1970’s for the first time [83]. Nowadays, superparamagnetic iron oxide nanoparticle-based MRI contrast agents are used in clinical applications [84]. Further, iron oxide based nanoparticles are in focus of research for their application as MRI contrast agents, including Fe_3_O_4_, MnFe_2_O_4_, as well as MnO [7,85–87]. Inspired by the progress for single component nanoparticles in the field of bioimaging, hetero-structured nanoparticles, such as Au@Fe_3_O_4_, moved into focus for future use as multimodal contrast agents [88–89]. For instance, PEG-functionalized Au@MnO “nanoflowers” were shown to combine optical and magnetic properties and, therefore, to be suitable for dual imaging [58]. Cu@Fe_3_O_4_ as well as Co@Fe_2_O_3_ combine magnetic and optical properties useful for simultaneous optical and magnetic imaging. Additionally, the magnetic properties may be enhanced due to the interaction at the nano-interface as shown for the exceptionally large T*_2_*-relaxation times of Co@Fe_2_O_3_ as compared to commonly available iron based MRI agents [59]. The most common metal nanoparticles for optical imaging with a long history are gold nanoparticles owing to their strong surface plasmon resonance. Additionally, the gold nanoparticles exhibit a strong X-ray absorption, which can be used to increase the contrast in CT diagnostics [90], as shown by the combination of gold nanoparticles with iron oxide to create multifunctional hetero-nanoparticles for simultaneous MRI and CT imaging [66,91].

**Figure 14:**
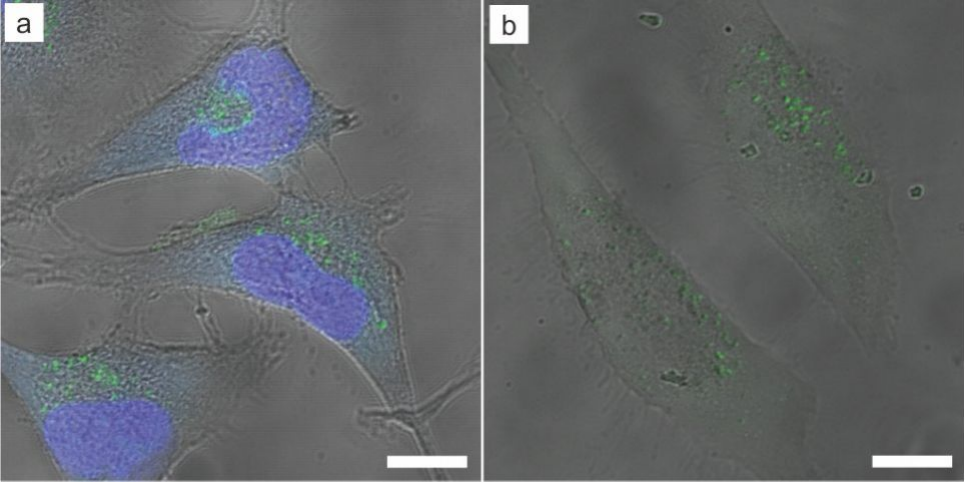
CLSM images of HeLa cells co-incubated with Au@MnO@SiO_2_-Atto495 Janus particles (green) for 24 h at 37 °C (c(Mn^2+^) = 100 µg/mL). a) λ*_ex_* = 488 nm, cell nuclei were stained using DAPI, b) two-photon image of the same sample, λ*_ex_*(2P) = 970 nm. Scale: 10 µm. Adapted with permission from [39]. Copyright 2014 American Chemical Society.

Moreover, Au nanorods as well as Au@MnO@SiO_2_ Janus particles were shown to emit strong photoluminescence under two photon excitation used for in vitro imaging (Figure 14) [39,92]. In comparison to conventional microscopy, multi-photon microcopy is superior regarding the reduced fluorescence background based on the little two photon-cross-section of most biomolecules leading to less auto-fluorescence, enhanced penetration depth within biological samples by tuning the excitation light to the biological window, near IR range 700–1000 nm. Furthermore, the effect of photobleaching can be reduced by selective excitation of the focal volume [93–94]. Nevertheless, it remains challenging to extend the technique to time-dependent measurements for clinically relevant volumes, reaching beyond small animals used as testing systems at the moment [95–98].

### Surface modification of Janus particles

The synthesis of monodisperse, well-defined hetero-nanostructures requires non-hydrolytic reaction conditions, as established for the isotropic analogues. Therefore, it is necessary to exchange the hydrophobic surface functionalization of the nanoparticles by hydrophilic ligands to secure colloidal stability in an aqueous environment. This is a key point regarding the use of nanoparticles for biomedical applications for sensing biomolecules, cells, and diagnosis of diseases, and intracellular delivery [99–101]. There are different surface modification strategies, such as ligand exchange to bind bifunctional or multi-dentate ligands/polymers or the formation of amphiphilic micelles while maintaining the original hydrophobic ligand shell. However, all these strategies suffer from the assumption that the ligand coating is neither densely packed nor static with regards to ligand exchange when the particles are diluted in biological media [102]. On the contrary, the encapsulation of isotropic nanoparticles in a silica shell was established, which is advantageous because of the extraordinary stability of silica and its well-known surface chemistry that allows further functionalization. Furthermore, the silica shell preserves the intrinsic materials properties, such as magnetic or plasmonic characteristics, but it minimizes the release of toxic ions from the nanoparticle surface and a direct contact with cells. Due to its higher packing density as compared to an organic ligand coating, it also prohibits the diffusion of water or solvent molecules to the surface of the underlying particle [103].

**Scheme 1:**
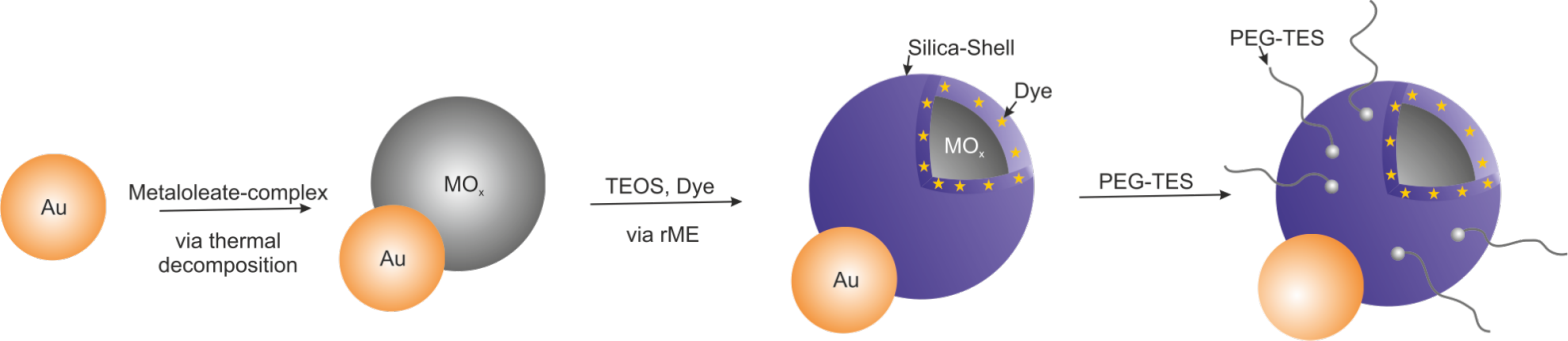
Seed-mediated synthesis of Au@MO_x_ heterodimers, subsequent encapsulation with silica and functionalization of the SiO_2_-shell. Adapted with permission from [39]. Copyright 2014 American Chemical Society.

The hydrophobic particles are encapsulated using the reverse-microemulsion technique, which can be applied to a large variety of core materials [101,104–108]. Consequently, this method can easily be transferred to Janus particles: Independently, which metal oxide was grown on gold seeds, the metal oxide domain could be encapsulated selectively by SiO_2_ using a reverse microemulsion technique (Scheme 1) [38–39].

**Figure 15:**
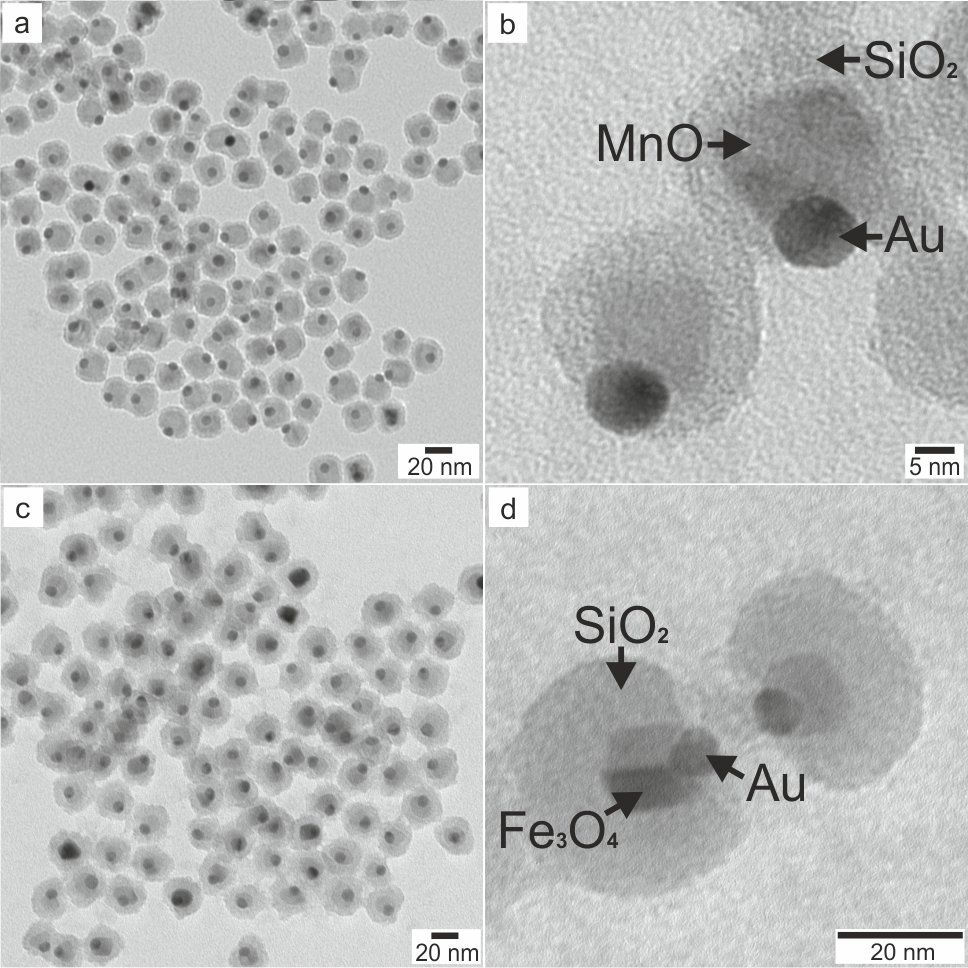
TEM micrographs of silica encapsulated Janus particles; (a,b) Au@MnO@SiO_2_ (10@20 nm), and (c,d) Au@Fe_3_O_4_@SiO_2_ (9@15 nm).

Due to the different chemical wetting behavior of gold and the metal oxide surface, only the metal oxide domain is encapsulated leaving the hydrophobic character of the gold domain untouched (Figure 15). This selectivity is enhanced due to the functionalization of the gold domain with a thiol, which can be performed either prior to the growth of manganese oxide or after formation of the iron oxide component.

**Figure 16:**
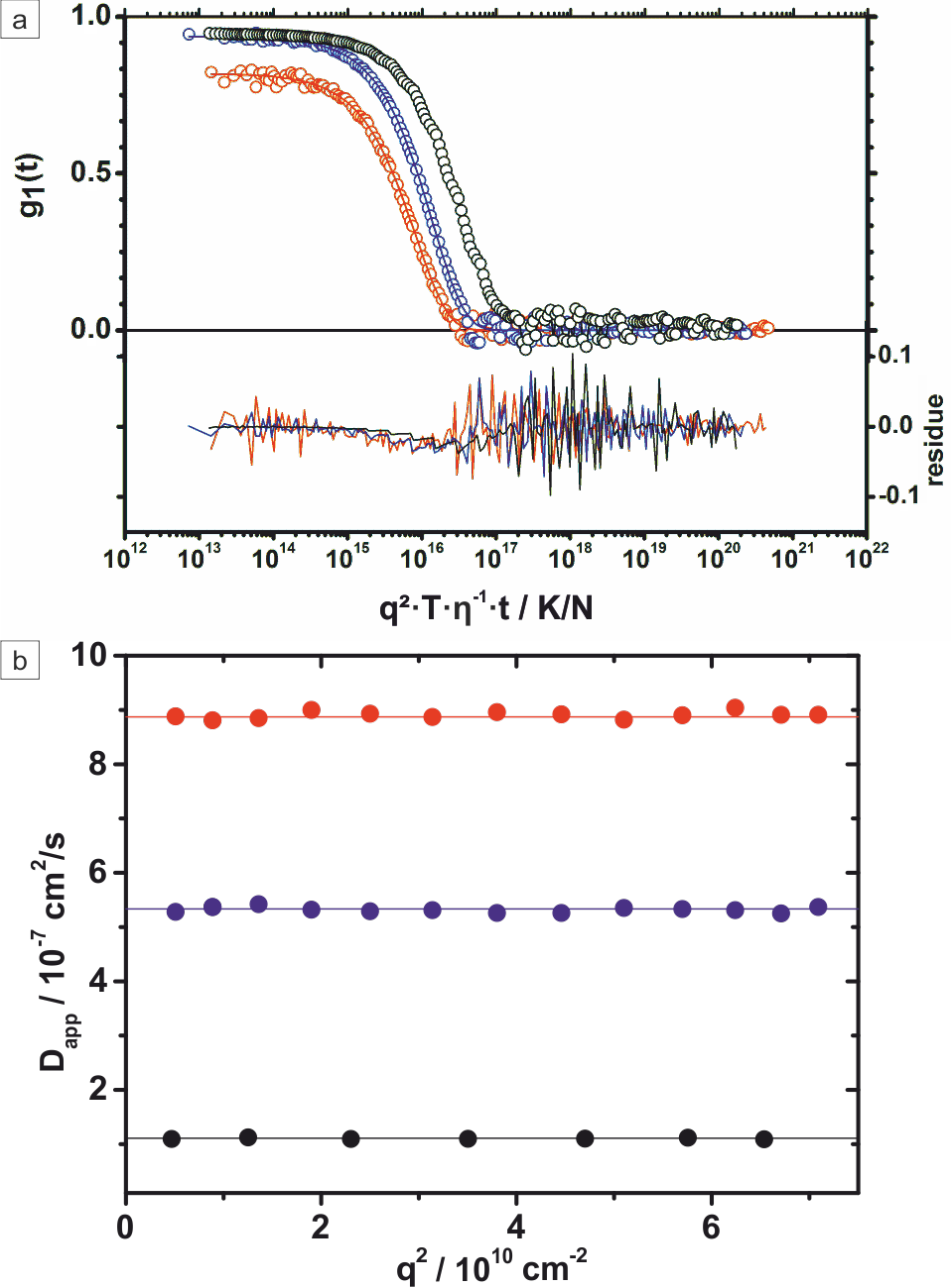
Dynamic light scattering results of Au (red dots), Au@Fe_3_O_4_ (blue dots) dispersed in n-heptane, and Au@Fe_3_O_4_@SiO_2_ (black dots) in water (λ = 632.8 nm, *T* = 293 K, viscosity η: 0.41 cP n-heptane, 1.005 cP water). a) Universally scaled autocorrelation functions measured at scattering angle θ = 30° together with biexponential fitting function lines and corresponding residues. b) Apparent diffusion coefficients as a function of the scattering vector q^2^ in the range of scattering angle 30° ≤ θ ≤ 150°.

Wu et al. pointed out that a thiol passivation of the surface is crucial for retaining the Janus character due to the two different surfaces [38]. This was demonstrated by complete encapsulation of Au@Fe_3_O_4_ as well as Ag@Fe_2_O_3_ nanoparticles with a silica shell [103]. The stability of the silica coated particles against aggregation and biocompatibility is enhanced by surface modification using a PEG-silane conjugate. Therefore, the particles remain stable at all stages of the preparation as confirmed by DLS measurements of hydrophobic Au nanoparticles, Au@Fe_3_O_4_ heterodimers, and silica-encapsulated Au@Fe_3_O_4_@SiO_2_ Janus particles, the results of which are shown in Figure 16. As apparent from the amplitude of the autocorrelation function fits and the constancy of the apparent diffusion coefficients *D*_app_ as a function of the scattering angle θ, all three samples are highly monodisperse with sizes of *R*_h_(Au) = 6.5 nm, *R*_h_(Au@Fe_3_O_4_) = 12.8 nm, and *R*_h_(Au@Fe_3_O_4_@SiO_2_) = 19.5 nm. The progressive increase of the hydrodynamic radii is in good accordance with the increase in particle diameter. The thickness of the silica shell was determined by TEM, and matches the expected contribution of the hydration shell in non-polar or aqueous solution.

**Figure 17:**
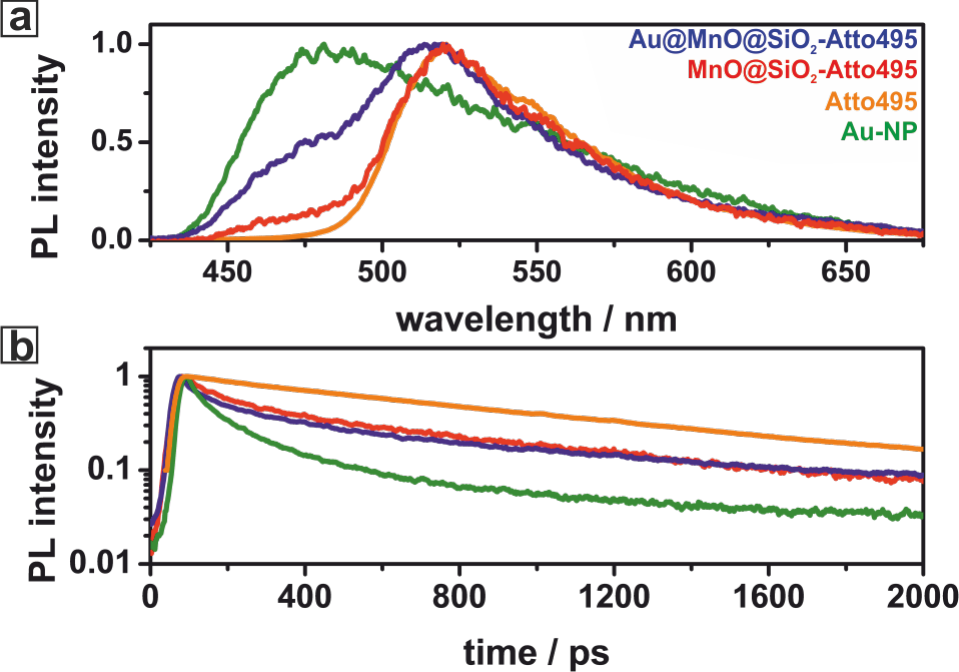
(a) Time-resolved fluorescence spectra of Au nanoparticles (green), Atto495 (orange), MnO@SiO_2_ (red), and Au@MnO@SiO_2_-Atto495 (blue) after excitation at λ = 400 nm by a 100 fs pulse indicating the absence of electron transfer between Au and MnO respectively Atto495 encapsulated within the SiO_2_ shell; (b) respective photoluminescence dynamics tracked at 500–550 nm.

Time-resolved photoluminescence measurements showed that the photoluminescence dynamics of Au@MnO@SiO_2_-Atto495 Janus particles can be described by a bi-exponential decay progress. That is, it consists of two independent exponential decay processes, namely the two individual photoluminescence decays of Au and MnO@SiO_2_-Atto495 [39] (Figure 17). However, the fluorescence of Atto495 is quenched partially when bound to MnO@SiO_2_ and the resulting photoluminescence decay described by a bi-exponential function yields the photoluminescence lifetime equivalent to that of pristine Atto495 and a second, but reduced PL lifetime as a consequence of the metal oxide-dye interaction.

Additionally, aminosilanes, such as *N*-(6-aminohexyl)aminopropyltrimethoxysilane (AHAPS) or (3-aminopropyl)trimethoxysilane (APS), were applied to control the surface charge by introducing amino-groups at the surface [108–109]. The resulting positively charged nanoparticles are known to be taken up more efficiently in in vitro cultures [110–111], whereby amine-functionalized silica-particles enable covalent conjugation of dyes, biomolecules, such as sugars, antibodies, and peptides [112]. Consequently, this synthetic route allows precise control over nanoparticle composition, domain sizes, surface functionalization, and, also, surface charge.

### Biomedical applications

**Figure 18:**
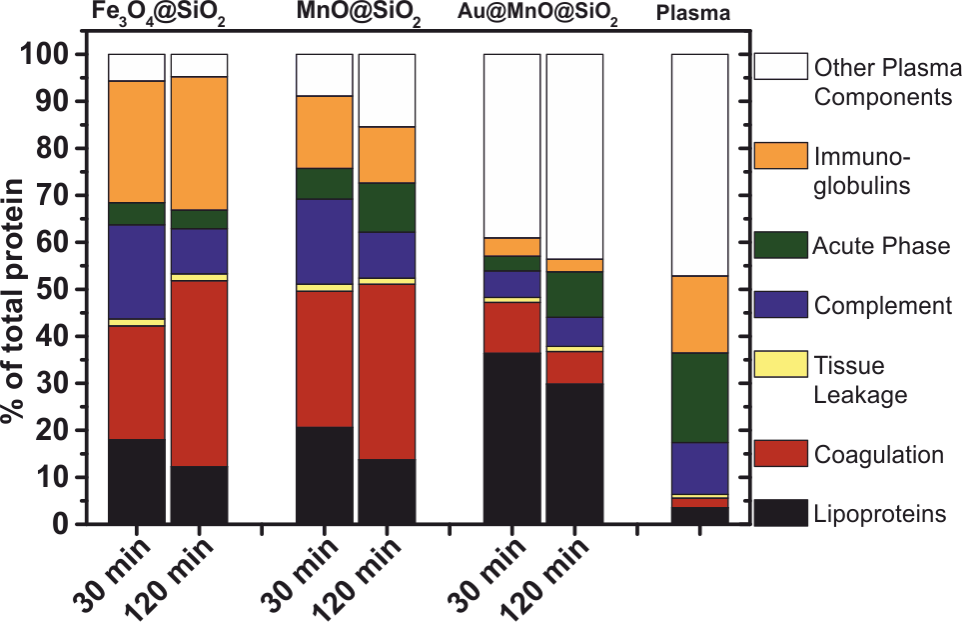
Labelfree LC-MS Analysis of the hard protein corona of Fe_3_O_4_@SiO_2_, MnO@SiO_2_, and Au@MnO@SiO_2_ nanoparticles showing a dependence on composition, morphology, as well as incubation time.

When nanoparticles get in contact with body fluids, a variety of serum components binds to the surface of nanoparticles, while the composition of the protein corona is responsible for the fate of the nanoparticles in the organism. Up to now, the formation and the composition of the protein corona is far from being completely understood, but the importance is obvious for any further application of nanoparticles in theranostics [113]. The analysis of the protein corona of nanoparticles shows that the binding profiles do not reflect the relative protein concentrations of the plasma. Recently, Tenzer et al. showed that there is no direct correlation of the surface charge and the isoelectric point of proteins enriched in the protein corona of silica nanoparticles. Moreover, no size-dependent particle-protein binding effect was observed while studying nanoparticles with a diameter of 125 nm, 20 nm, and 8 nm [114]. Introducing the anisotropy of Janus particles as another variable to the formation/analysis of the protein corona increases the complexity even more. Figure 18 shows a comparison of the composition of the protein corona for isotropic Fe_3_O_4_@SiO_2_ and MnO@SiO_2_ as well as anisotropic Au@MnO@SiO_2_ Janus particles. As was observed for pure SiO_2_ nanoparticles [114], a significant enrichment of lipoproteins and proteins involved in coagulation as compared to plasma was measured. The amphiphilic Janus character is reflected in the specific protein adsorption pattern. It shows a distinct enrichment of lipoproteins and other plasma components to the isotropic analogues confirming the observation of preferentially binding of apolipoproteins and serum albumin to hydrophobic nanoparticles by Cedervall and co-workers [115]. Interestingly, there is a significant and unexpected difference in the composition of the protein corona of isotropic silica encapsulated MnO and Fe_3_O_4_ nanoparticles. By studying the protein adsorption to silica particles of varying sizes and surface functionalization, it could be shown that small changes also are sufficient to drastically change to the affinity for peptides [116–118]. As a consequence, it is possible that slight structural differences of the silica shell of MnO and Fe_3_O_4_ nanoparticles due to the underlying nanoparticles lead to significant changes in protein interaction.

## Conclusion

Although Janus particles were established conceptually only about 20 years ago, a large body of research has been invested in this field of colloidal chemistry. In this review, we highlighted important synthetic methods focusing on heterogeneous nucleation, which facilitates the control of size and morphology of particles varying from dumbbell-like structures to nanoflowers or core/shell structures. Recent synthetic developments have made promises for applying Janus particles due to their outstanding properties, even at large scale.

Janus particles combine two or more distinct components in chemistry, properties, and morphology. Such particles exhibit many intriguing properties, including amphiphilic, magnetic, optical, and catalytic characteristics, therefore, opening a wide range of potential applications as catalysts, in drug delivery, biomedical imaging, high-throughput immunoassays, for biological probing, and remote manipulation of devices. In addition, Janus particles may find use as surfactants, water-repellent coatings, or building blocks for supramolecular structures. We put emphasis on dumbbell-like structures composed of a metal- and a metal oxide-domain. The chosen materials exhibit several unique advantages useful for biomedical applications, such as for theranostic agents. The metal domain, in most cases gold or platinum, were chosen based on their strong plasmonic resonance and their additional strong contrast for CT-analysis, which enables dual detection for in vivo analysis. Furthermore, the materials of the metal oxide domain were chosen to exhibit magnetic behavior to be able to apply the multifunctional Janus particles as MRI contrast agents. As long term stability in aqueous media is of particular importance for biomedical applications, the extraordinary stability of silica encapsulated nanoparticles and the well-known surface chemistry of silica were transferred to Janus particles, whereby the Janus character was retained due to the distinct chemical surface characteristics of the chosen materials.

Although, Janus particles were demonstrated to be a powerful tool for widespread applications, several challenges have to be overcome. Therefore, the following aspects might find particular attention in the future. Janus particles can be purely inorganic, purely polymeric, and hybrid-type inorganic-polymeric. The inorganic and polymeric components can differ in both chemical composition and morphology. Therefore, a large library of Janus particles with various chemical compositions and morphologies can be made in theory from the available components. Our current fabrication methods do not allow the synthesis of Janus particle libraries for a systematic screening of the particle properties. Therefore, synthetic efforts must be made to develop reliable strategies for making Janus particles at large scale.

Many Janus particles reported so far are fairly simple systems. They may be viewed as test cases for developing new synthetic methods. Janus particles with practical applicability can be envisioned by integrating specific functions, such as superparamagnetism, fluorescence, biocompatibility, or catalysis, stimulus-responsiveness, and dispersibility in various media.

Hybrid inorganic-polymeric Janus particles are expected to have different, complementary properties compared to their purely inorganic and purely polymeric counterparts. The interactions between those components may be tuned or reversed easily. This field appears to have the largest potential for development.

The self-assembly of colloidal Janus particles may lead to the formation of complex hierarchical structures that do not emerge spontaneously as thermodynamically stable assemblies when particles are mixed. Fundamental studies that can help understanding the principles of self-assembly are highly valuable.

In summary, it could be shown that these particles already show a large variety compared to other fields of colloidal chemistry, although, this area is only burgeoning. New and powerful methods for the preparation of Janus particles will have to be developed to allow an exploration of their physicochemical properties and large scale applications in the near future.
